# Molecular Characterization of Pediatric Restrictive Cardiomyopathy from Integrative Genomics

**DOI:** 10.1038/srep39276

**Published:** 2017-01-18

**Authors:** Tara N. Rindler, Robert B. Hinton, Nathan Salomonis, Stephanie M. Ware

**Affiliations:** 1 The Heart Institute Cincinnati Children’s Hospital, Cincinnati, Ohio, U.S.A; 2 Biomedical Informatics Cincinnati Children’s Hospital, Cincinnati, Ohio, U.S.A; 3 Department of Pediatrics and Medical and Molecular Genetics, Indiana University School of Medicine, Indianapolis, Indiana, U.S.A

## Abstract

Pediatric restrictive cardiomyopathy (RCM) is a genetically heterogeneous heart disease with limited therapeutic options. RCM cases are largely idiopathic; however, even within families with a known genetic cause for cardiomyopathy, there is striking variability in disease severity. Although accumulating evidence implicates both gene expression and alternative splicing in development of dilated cardiomyopathy (DCM), there have been no detailed molecular characterizations of underlying pathways dysregulated in RCM. RNA-Seq on a cohort of pediatric RCM patients compared to other forms of adult cardiomyopathy and controls identified transcriptional differences highly common to the cardiomyopathies, as well as those unique to RCM. Transcripts selectively induced in RCM include many known and novel G-protein coupled receptors linked to calcium handling and contractile regulation. In-depth comparisons of alternative splicing revealed splicing events shared among cardiomyopathy subtypes, as well as those linked solely to RCM. Genes identified with altered alternative splicing implicate RBM20, a DCM splicing factor, as a potential mediator of alternative splicing in RCM. We present the first comprehensive report on molecular pathways dysregulated in pediatric RCM including unique/shared pathways identified compared to other cardiomyopathy subtypes and demonstrate that disruption of alternative splicing patterns in pediatric RCM occurs in the inverse direction as DCM.

Pediatric cardiomyopathy is a clinically and genetically heterogeneous form of heart muscle disease classified into five clinical subtypes: hypertrophic cardiomyopathy (HCM), dilated cardiomyopathy (DCM), restrictive cardiomyopathy (RCM), left ventricular noncompaction cardiomyopathy (LVNC), and arrhythmogenic right ventricular cardiomyopathy (ARVC)^1^. Approximately 40% of symptomatic pediatric cardiomyopathy patients will require a heart transplant or die within five years of diagnosis. RCM is characterized by diastolic dysfunction in the absence of cardiac dilation, hypertrophy or significant systolic dysfunction, commonly described as an abnormality of right or left ventricular filling. Patients with RCM, the rarest form of cardiomyopathy (2–5%), have the poorest prognosis with high mortality in the near term leading to the current recommendation of cardiac transplantation at the time of diagnosis^2^. The lack of effective medical treatment for children with this disease highlights the need to better understand molecular factors affecting its development and progression.

The genetic basis of RCM remains largely unknown although mutations in sarcomeric genes that can also cause HCM have been identified as causative in a subset of affected patients^3^. However, even within families with a known genetic cause for cardiomyopathy there is striking variability in the phenotype, age of onset, and disease severity^4^. This phenotypic variation suggests the presence of major genetic modifiers and/or activation of multiple genetic pathways that additively contribute to the phenotype. The primary molecular pathways dysregulated in RCM are poorly understood. In contrast, DCM and heart failure in the adult population have been molecularly characterized. These reports identify heart failure by its distinct mRNA splicing patterns, including several reports that suggest activation of embryonic splicing patterns in diseased hearts^5^,^6^. Recent reports implicate a RNA-binding protein (RBM20) in DCM progression. RBM20 is required for alternative splicing of several genes associated with cardiomyopathy including: titin (*TTN*), ryanodine receptor 2 (*RYR2*), calcium/calmodulin-dependent protein kinase II delta (*CAM2D*), and LIM domain binding 3 (*LDB3*)^7^,^8^; furthermore, decreased levels of RBM20 in DCM leads to retention of commonly spliced exons. To our knowledge, no previous studies have examined gene expression or alternative splicing data for pediatric RCM.

The goal of the present study was to define patterns of gene regulation in RCM using healthy non-failing hearts as controls and adult heart failure hearts as comparisons. We hypothesize that the expression profile of pediatric RCM will display characteristic molecular signatures specific to disease, and furthermore facilitate identification of potential novel therapeutic targets for the treatment of RCM. Our results uncover a largely novel set of transcriptionally regulated genes underlying cardiomyopathy in general and RCM specifically. These results extend to alternative splicing, where we identified RBM20 as a potential mediator of alternative splicing in RCM.

## Results

### RCM Subjects

All patients had an autosomal dominant inheritance pattern of RCM within the family. The molecular cause of RCM was identified in 3 of 4 subjects (Table 1). All four subjects had clinical genetic testing for cardiomyopathy as part of their clinical evaluation. Only subject RCM3 had a pathogenic mutation identified by clinical testing. Subsequently, RCM1 and RCM2, who are siblings, had research based testing performed using whole exome sequencing (Tariq *et al*., manuscript in preparation). All subjects fulfilled the following diagnostic criteria by echocardiography: one or both atria enlarged relative to normal or small sized ventricles with evidence of impaired diastolic filling and normal measures of posterior wall thickness (PWT), end-diastolic septal thickness (LVED), left ventricular mass (LVM) and left ventricular systolic function (FS). Constrictive pericarditis and significant valvar heart disease were absent in each patient. In addition, cardiac catheterization demonstrated elevated atrial pressures and restrictive physiology (Table 2).

### Differential Gene Expression

**Figure 1:**
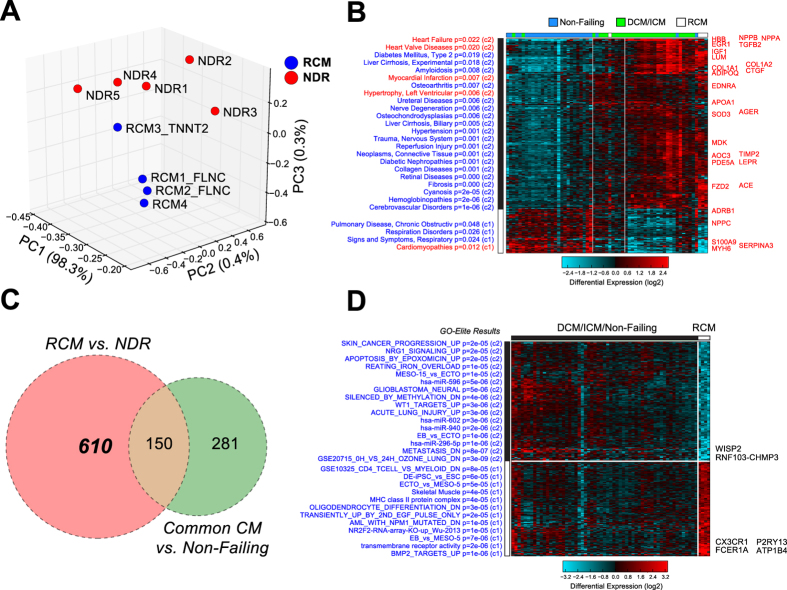
Differential Gene Expression in Pediatric Restrictive Cardiomyopathy. (**A)** Principal component (PC) analysis using Single Value Decomposition and z-score expression normalization of RCM samples (blue) and control samples (red). The different visualized axes represent the separation of samples according to each the respective top 3 PCs. The percentage of associated variance described by each PC is shown. (**B)** Heatmap depicting common differential expression of genes in cardiomyopathy (RCM and ICM/DCM datasets (GSE48166 and GSE55296)) verses non-failing hearts, following moderated FDR adjusted t-test p < 0.05, and a fold change > 1.5. RCM samples are highlighted with white above the cardiomyopathy cluster. Pathway enrichment analysis was performed for each gene cluster using the disease ontology analysis, with cardiac ontology terms and corresponding genes highlighted in red. (**C)** Venn diagram identifying RCM specific genes. RCM versus NDR controls (pink) identifies the RCM regulated genes. A Common Cardiomyopathy comparison (cardiomyopathy versus non-failing (green)) identifies cardiomyopathy specific genes. (**D)** Heatmap depicting 359 RCM specific genes, identified by filtering the 610 RCM specific genes to exclude genes identified by the comparison of RCM to the union of all non-RCM samples (moderated FDR adjusted t-test p < 0.05). Pathway enrichment analysis was performed for each gene cluster using the disease ontology analysis, with the most significantly up- and down-regulated genes listed to the right of the heatmap.

As an initial evaluation of this data, we performed principal component analysis (PCA) using Single Value Decomposition with z-score normalization and analyzed the top 100 loading genes associated with the top three principal components (correlated and anticorrelated) (Fig. 1A). Although over 80% of the total variance in this dataset could be explained by the first PC (PC1), the associated PC1 loading genes were selectively expressed in the different individual patient samples. However, PC3 ordered samples and genes (hierarchical clustering) segregated the samples according to disease status (Supplemental Figure S1). The PCA of our dataset demonstrated clustering of RCM samples (blue) and NDR control samples (red) into distinct subsets. Comparison of RCM versus NDR (controls) yielded 760 differentially expressed genes with at least a 1.5 fold difference and moderated t-test p < 0.05. To further control for possible differences in age or cardiac sample site, we analyzed previously described datasets profiling non-diseased human left and right ventricles (GSE57338 and GSE36761; See Table 3 for description of all datasets)^9^,^10^. We identified only a small number of genes anti-correlated with age (n = 10), none of which were increased in RCM (GSE57338). Additionally, we were unable to find any common genes regulated in our RCM analysis with a similar or greater magnitude when comparing non-diseased left ventricle and paired right ventricle RNA-Seq samples (GSE36761) (data not shown). These data suggest that RCM-associated differences identified in this study are likely due to disease rather than donor age or ventricular sample site.

There are several reports on the molecular pathways that become dysregulated during DCM and heart failure in adults. To determine which gene expression differences in RCM are due to common regulatory pathways impacted in cardiomyopathies, we analyzed two additional human RNA-Seq datasets. The first is from subjects with adult idiopathic cardiomyopathy (ICM) and non-failing control patients (GSE48166, GEO, Table 3). This data set, which contains heart transcriptomes on 15 ICM and 15 control hearts obtained at the time of transplantation, identified over 1,000 differentially expressed genes. Another recently reported dataset on ICM and DCM (GSE55296, GEO)^11^ contains complete RNA-Seq data on 24 human samples (8 ICM and 8 DCM compared with 8 non-diseased donor hearts). The DCM patients had impaired left ventricular systolic function accompanied by ventricular dilation on echocardiography, and the ICM patients were further characterized by coronary angiography data to identify cardiac ischemia^11^. For consistency, we re-analyzed these datasets using the same sequence alignment and quantification methods (RNA-Seq by Expectation Maximization, RSEM).

**Figure 2:**
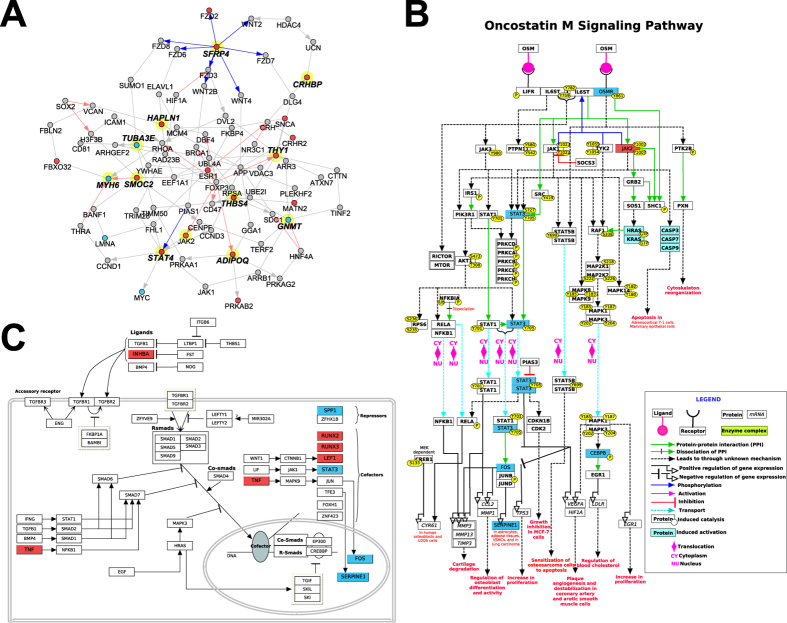
Network and Pathways of Cardiomyopathy Regulated Genes. (**A)** Network of Common Cardiomyopathy regulated genes. Genes commonly upregulated or down-regulated in multiple cardiomyopathy datasets, including RCM, are shown. Genes were connected via protein-protein, pathway, or ChIP-Chip interactions from the NetPerspective algorithm in AltAnalyze. Genes connected by the shortest path among the 14 commonly regulated genes (bold text) are shown, with those up-regulated in RCM vs. non-failing hearts in red and those down-regulated in blue. (**B)** Oncostatin M (OSM) pathway, highlighting the downstream regulation of the janus kinase/signal transducer and activator of transcription (JAK/STAT) pathway, with the pathway components up-regulated in RCM vs. non-failing hearts in red and those down-regulated in blue. (**C**) Transforming growth factor beta (TGFβ) signaling pathway highlighting activation of tumor necrosis factor-alpha (TNF), with the pathway components up-regulated in RCM vs. non-failing hearts in red and those down-regulated in blue.

To generate a Common Cardiomyopathy genetic profile, the cardiomyopathy dataset (RCM, DCM, and ICM) was compared to non-failing hearts (Table 3) following a moderated FDR adjusted t-test p < 0.05, and a fold change > 1.5 (Fig. 1B). We identified 431 differentially expressed genes in the Common Cardiomyopathy dataset (green circle, Fig. 1C). Furthermore, our RCM dataset demonstrated a consistent pattern of gene expression (induction and repression) when compared to the other cardiomyopathies (RCM data columns are highlighted in white above the cardiomyopathy cluster, Fig. 1B). Pathway enrichment analysis was performed for each gene cluster using disease ontology gene enrichment analysis. Several cardiac disease ontology terms were identified including: heart failure, heart valve disease, myocardial infarction, left ventricular hypertrophy, and cardiomyopathy. These disease ontology terms include the following genes: plasminogen activator inhibitor-1 (*SERPINE1*) which has previously been shown to be involved in myocardial remodeling following ischemia reperfusion injury^12^, the well characterized cardiomyopathy associated α-myosin heavy chain 6 (*MYH6*)^13^, and several genes from the natriuretic peptide (NP) system which have well-known roles as biomarkers in heart failure (*NPPA, NPPB*, and *NPPC*)^14^. Furthermore, network analysis of cardiomyopathy-regulated genes identified genes connected among the 14 most commonly regulated genes in our dataset, including secreted frizzled-related protein 4 (*SFRP4*), hyaluronan and proteoglycan link protein 1 (*HAPLN1*), alpha tubulin 3e (*TUBA3E*), SPARC related modular calcium binding 2 (*SMOC*2), signal transducer and activator of transcription 4 (*STAT4*), adiponectin CQ (*ADIPOQ*), thrombospondin 4 (*THBS4*), thy-1 cell surface antigen (*THY1*), glycine N-methyltransferase (*GNMT*), corticotropin releasing hormone binding protein (*CRHBP*), and *MYH6* (Fig. 2A). Comparison of genes with shared upregulation in the Common Cardiomyopathy dataset found 11 shared upregulated genes (*SMOC2, ADIPOQ, STAT4, THBS4, THY1, ASPN, CRHBP, SFRP4, CILP, OGN*, and *HAPLN1*) and 3 down-regulated (*GNMT, TUBA3E*, and *MYH6*). Intriguingly, only a few of these (*MYH6, ADIPOQ, STAT4*, and *THY1*), have been previously reported to be generally associated with cardiomyopathy to our knowledge^13^,^15^,^16^,^17^.

To identify genes specifically dysregulated in RCM, we compared genes differentially expressed in NDR vs RCM (pink circle, Fig. 1C), as well as the Common Cardiomyopathy dataset (green circle, Fig. 1C). This produced a set of 610 genes, up- and down-regulated in RCM but not in DCM or ICM. We further filtered these 610 genes down to a set of 359 genes that remain significant when comparing RCM to union of all non-RCM samples from Fig. 1B (moderated FDR adjusted t-test p < 0.05). We found that these up- and down-regulated genes were enriched in a diverse set of biological pathways (Fig. 1D). These included transcriptional targets of nuclear receptor subfamily 2F2 (*NR2F2*) and bone morphogenetic protein 2 (*BMP2*), suggesting repression of these transcriptional regulators. Given that RCM results in diastolic versus systolic dysfunction, we focused on genes associated with calcium handling and regulation of contraction/relaxation. Genes associated with elevation of cytosolic calcium ion concentration (*CX3CR1, GLP1R, AVPR1A*, and *PTGER3*) were statistically enriched among upregulated RCM genes, all of which are members of the G-protein couple receptor family. G-proteins that inhibit adenylate cyclase (Gi) can modulate cardiac contraction^18^. Additionally, arginine vasopressin receptor 1A (*AVPR1A*) was previously shown to module heart contraction and heart failure^19^, and CX3C chemokine receptor 1 (*CX3CR*1), and Prostaglandin E receptor 3 (*PTGER3*) are all indirectly linked to contractile defects in the heart via regulation of the Fractalkine pathway^20^. Furthermore, the glucagon-like peptide 1 receptor (*GLP1R*) has previously been linked to diastolic dysfunction and ventricular hypertrophy^21^. Among the most significantly upregulated genes in RCM was the recently discovered GPCR P2Y purinoceptor 13 (*P2RY13*) which is also coupled to the Gi-signaling pathway^22^. Importantly, these gene expression profiles are specific to RCM and not found to be dysregulated in DCM.

Since it is possible that RCM regulation is in an opposing direction to that previously observed in ICM or DCM, we specifically looked for such cases (Supplemental Table 1). Only one gene was inversely regulated in RCM compared to the Common Cardiomyopathy dataset: SEC14-like 5 (*SEC14L5*), an integral membrane protein with predicted transporter activity. A small number of other genes were regulated inversely in different adult forms of cardiomyopathy (RCM/DCM or RCM/ICM datasets) relative to RCM, including intelectin 1 (*ITLN1*), delta-like 1 homolog (*DLK1*), reelin (*RELN*), and myosin light chain kinase family, member 4 (*MYLK4*). A previous study confirmed downregulation of *MYLK4* in ICM, whereas this gene is upregulated in RCM^23^. Furthermore, several pathways previously shown to play a role in the development of heart failure are differentially dysregulated in pediatric RCM including; janus kinase/signal transducer and activator of transcription (JAK/STAT) pathway (*JAK2, STAT3, MYC, OSMR*, and *IL4R*) (Fig. 2B), and transforming growth factor beta (TGFβ) pathway (*INHBA, TNF, RUNX2, RUNX3, LEF1*, and *STAT3*) (Fig. 2C)^24^,^25^. This dysregulation of the JAK/STAT pathway results in decreased *STAT3* expression, accompanied by increased *JAK* expression (Fig. 2B). It has been proposed that decreased expression of *STAT3* in diseased hearts may contribute to the progression of heart failure,^24^,^26^ further suggesting that the dysregulation of the JAK/STAT pathway may contribute to the pathogenesis of RCM.

It has been well established that there is a general induction of the cardiac fetal gene program in the failing heart^27^,^28^. However, in our RCM data-set there was not a clear pattern of fetal gene reprograming. Only two fetal genes were upregulated in RCM (*HAPLN1* and *TNNI1*)^29^,^30^, and one fetal gene was downregulated (*MYH6*). Taken together, these results highlight the unique genetic profile of pediatric RCM compared with adult forms of cardiomyopathy.

### Analysis of Alternative Splicing

**Figure 3:**
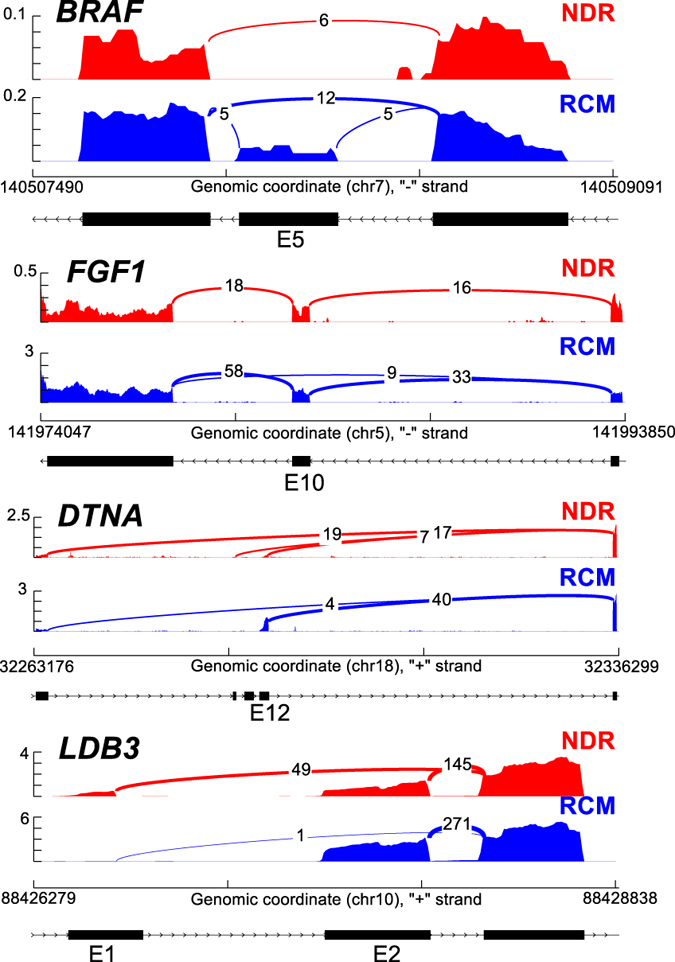
Sashimi Plots of alternative exon and promotor usage. Sashimi plots of alternative splicing of *BRAF* and *FGF1* depicting exon inclusion (exon 5, E5, and exon 10, E10, respectively), and *DTNA* and *LBD3* depicting alternative promotor usage.

In addition to gene regulation, RNA-Seq readily allows for the detection of known and novel alternative mRNA isoforms. Given that accumulating evidence suggests an important role for alternative splicing and splicing factor regulation in cardiomyopathy^5^,^6^,^7^,^8^,^31^, we performed an extensive analysis of alternative exon regulation in our RCM dataset as well as other adult cardiomyopathy datasets in our software AltAnalyze using the percent spliced in (PSI) method. Among the most statistically enriched alternatively spliced genes in NDR vs RCM were those associated with cardiomyopathies (*ANK2, CD36, DCN, DTNA, ITGAV, MYH7, SLC8A1*, and *TLR4*), ventricular outflow obstruction (*CD36, ENO3, MYH7*, and *PPP3CB*), regulation of heart contraction (*ANK2, CAMK2D, HRC, SLC8A1*, and *TPM3*), actin cytoskeleton (*CALD1, DST, PDLIM3, PEAK*1, *SEPT7, SVIL*, and *TPM3*) and Z-disk (*ANK2, DST, MYH7, OBSCN, PDLIM3, SLC8A1*, and *TTN*) (ToppGene). Sashimi plots^32^ depicting representative examples of alternative exon inclusion (*BRAF* and *FGF1*) and the use of alternative promotors (*DTNA* and *LBD3*) are shown in Fig. 3. We excluded two splicing events from this list that were also found in our control comparison of RCM with GSE36761 non-diseased left versus right ventricle (*SORBS2* and *ANK1*, out of 43 alternatively regulated genes).

**Figure 4:**
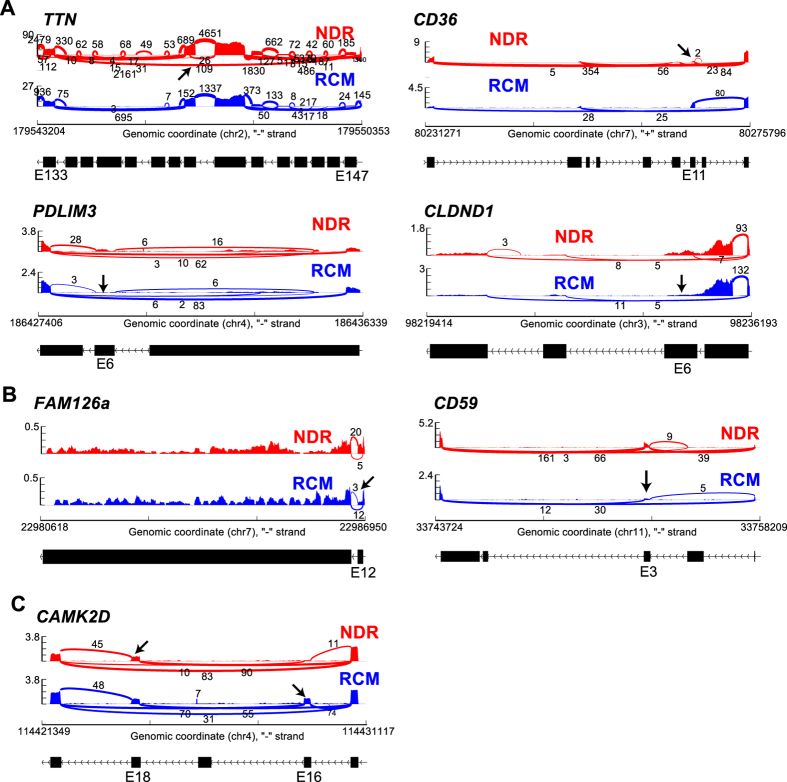
Sashimi Plots of alternative splicing. Sashimi plots of alternative splicing events (**A**) common to both RCM and the common cardiomyopathy dataset including *TTN, PDLIM3, CD*36, and *CLDND1*, (**B)** common between two datasets (RCM and ICM or RCM and DCM, relative to controls) including *FAM126a* and *CD59,* and (**C)** well-described RBM20 target *CAMK2D.*

Comparison of splicing events that were common to both RCM and the Common Cardiomyopathy dataset identified 4 events that were common to all (RCM, ICM, and DCM). For each of these comparisons, splicing was determined in each cardiomyopathy dataset compared to its own specific set of control samples. These include over 12 exons that were differentially spliced in titin (*TTN*) in cardiomyopathy samples, including exons 140 and 246, the co-occurring reduced inclusion of exon 6 of the cardiomyopathy associated protein PDZ and LIM domain protein 3 (*PDLIM3*)^33^, co-occuring increased inclusion of exon 11 in *CD*36, and inverse regulation of exon 6 in the tight junction protein claudin domain containing 1 (*CLDND1*)(AltAnalyze exon notations, Fig. 4A). The inclusion of *PDLIM3* exon 6 is predicted to result in nonsense-mediated decay in cardiomyopathy, while *CD36* exon 11 inclusion should result in protein truncation (AltAnalyze). Other similar splicing events were found in 12 distinct genes, common between the two datasets (RCM and ICM or RCM and DCM, relative to controls), but differing in the inclusion or exclusion junction, including family with sequence similarity 126, member a (*FAM126a*), toll-like receptor 4 (*TLR4*), and *CD59* (Fig. 4B). Splicing of exon 12 in *FAM126a* was found in both RCM and other forms of cardiomyopathy, but by distinct mechanisms (alternative 5′ splice site and cassette-exon inclusion, respectively).

**Figure 5:**
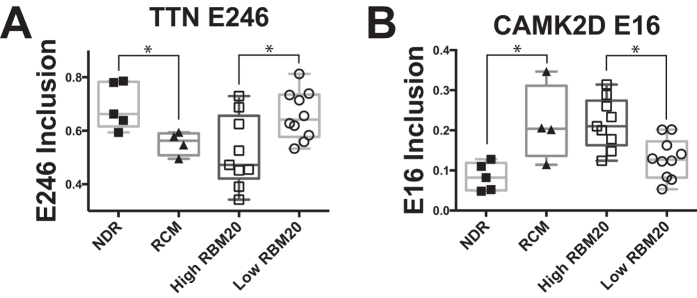
RBM20 regulated splicing events. Alternative splicing events for (**A**) titin (*TTN*) exon 246 inclusion, and (**B**) calcium/calmodulin-dependent protein kinase (*CAMKD2*) exon 16 inclusion in our RCM dataset and prior set of DCM patient samples with high and low RBM20 expression (E-MTAB-2572)^7^,^8^. Relative levels of exon inclusion are shown for the respective datasets. *p < .05

**Figure 6:**
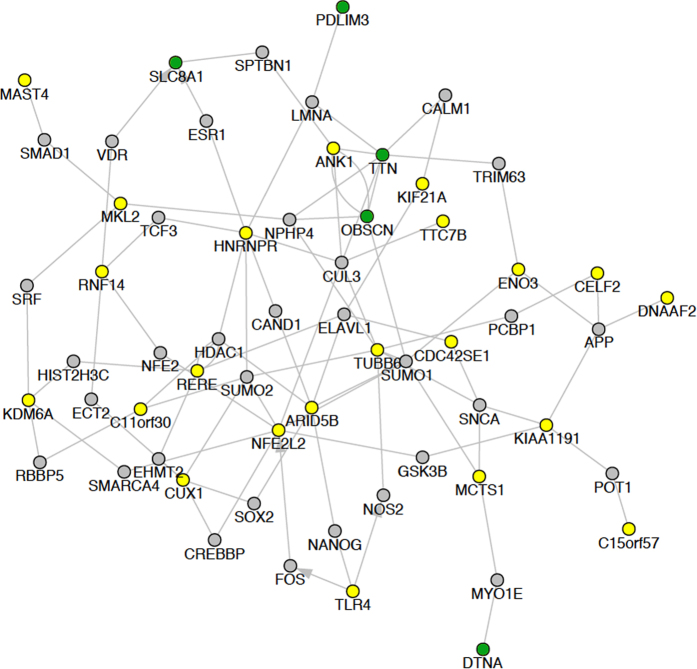
Network for most significant splicing events. Network analysis of splicing events with >25% difference, with a p-value < 0.05. The genes which display alternative splicing patterns in the RCM patients are depicted in yellow and green, with known targets of RBM20 highlighted in green.

We next analyzed splicing targets of RBM20, a well characterized splicing regulator that has been implicated in DCM progression^7^,^8^,^34^. To compare our splicing profiles to this prior set of patient samples with high and low RBM20 expression, we downloaded this RNA-Seq data (E-MTAB-2572) and compared calculated PSI values from this dataset to RCM^7^,^8^. Interestingly, the *TTN* splicing pattern observed in RCM (increased inclusion of exon 246) is in the opposite direction of that reported with low RBM20 (Fig. 5A)^7^,^8^. To test the hypothesis that RBM20-mediated splicing might be increased in RCM, we compared previously reported RBM20 predicted spliced targets in the dataset of high and low RBM20 expressing cardiomyopathies^7^,^8^. Interestingly, this analysis found the well-described RBM20 target, calcium/calmodulin-dependent protein kinase (*CAMKD2*), also mis-spliced in the inverse direction in our RCM dataset (Figs 4C and 5B). CAMK2D has been shown to undergo mutually exclusive splicing, which results in a coincident decrease of exon 16 and increase of exon 18 in patients with either decreased RBM20 expression or RBM20 mutation. In contrast to those results, we observed a significant increase in exon 16 inclusion (Figs 4C and 5B) and reciprocal decrease in exon 18 inclusion in *CAMKD2.* There is no significant difference in the gene expression levels of *TTN* and *CAMK2D* in our RCM data, suggesting that alternative splicing is the primary mode of regulation. Furthermore, several additional known targets of RBM20 showed splicing alterations within our data set (e.g. *PDLIM3, OBSCN*, and *DTNA*; Fig. 6) providing further evidence for RBM20 splicing abnormalities in RCM^7^,^8^. Taken together, these results support the hypothesis that RBM20 is playing a role in mediating alternative splicing patterns in pediatric RCM.

## Discussion

The critical modulators that affect presentation and compensatory mechanisms in cardiomyopathy are still poorly defined. Here, by analyzing pediatric RCM, in concert with previously characterized DCM and ICM samples, we have been able to distinguish common and disease-specific transcriptional networks. Our results indicate that both shared and distinct transcriptional dysregulation occurs in different cardiomyopathy phenotypes and demonstrate that alternative splicing alterations are common.

Analysis of pathways that were dysregulated across cardiomyopathy phenotypes identified shared genes that were associated with extracellular matrix remodeling and thus likely fibrosis (*THY1, CILP, OGN, THBS4, ASPN, HAPLN1,*and *SMOC2*), as well as calcium (*ADIPOQ, ASPN, THBS4, STAT4,* and *SMOC2*). Interstitial fibrosis is a common histological characteristic of the cardiomyopathies, which results in increased myocardial wall stiffness and alterations in excitation–contraction coupling^3^.

Identifying a unique transcriptional profile for the RCM phenotype is important for understanding disease mechanisms and identifying potential therapeutic targets. RCM specific expression was enriched among gene-sets implicated in positive regulation of cell death including (*TNF, CASP6, NFKBID, BCL2L11,*and *IRF6*), calcium signaling (*PHKG1, PTGER3, AVPR1A*, and *GRM1*), antigen processing and presentation (e.g., *CTSF, HLA-DPA1, HLA-DPB1, HLA-DQB2*), and transcriptional targets of forkhead box P3 (FOXP3; e.g., *NFKBID, CX3CR1, ITGAL,* and *IRF6*). Tumor necrosis factor (TNF) is a pro-inflammatory cytokine, which mediates diverse pathological processes in cardiac diseases, including ischemia, heart failure, and DCM^35^,^36^,^37^. Furthermore, previous studies demonstrated elevated expression of TNF in human DCM (undetectable in non-failing hearts), linking TNF expression to cardiac decompensation in DCM^35^. TNF has been shown to result in negative regulation of systolic function; additionally, cardiac TNF levels have been negatively correlated with disease severity^36^. Several studies suggest that the heart is capable of producing *TNF* mRNA in response to stress^37^. Inflammatory activation may be an important common component of cardiomyopathy and heart failure, yet it is becoming increasingly clear that distinct inflammatory pathways may be involved based on cardiomyopathy phenotype, chronicity, and underlying etiology^35^. Our results highlight specific patterns of dysregulation in RCM. Interestingly, in our RCM (RCM versus NDR) dataset we see a 3.5-fold increase in *TNF* mRNA expression (Fig. 2C), which maybe a contributing factor to the severity of the RCM phenotype as a recent report has implicated TNF signaling in cardiac fibrosis^38^. Additional mediators of cardiac fibrosis were dysregulated in RCM including several members of the transforming growth factor beta (TGFβ) pathway (*INHBA, TNF, RUNX2, RUNX3, LEF1*, and *STAT3*)^39^,^40^,^41^. Taken together, this suggests that the characteristic diastolic dysfunction of RCM may result from stiffening of the ventricles by cardiac fibrosis^42^.

Genes downregulated in RCM include those associated with adipogenesis (*SREBF1, GATA2, LMNA, GADD45B, CEBPB, STAT3,* and *SERPINE1*), janus kinase/signal transducer and activator of transcription (JAK/STAT) pathway (*STAT3, MYC, OSMR,* and *IL4R*), interleukin *(IL-2, IL-4,* and *IL5*) and brain-derived neurotrophic factor (*BDNF*) signaling. The JAK/STAT pathway mediates signal transduction from the plasma membrane to the nucleus. In the heart, the JAK/STAT pathway is activated by binding of interleukin (IL)-type cytokines to receptors which phosphorylate JAKs, leading to the subsequent phosphorylation of STATs^24^. As discussed above related to TNF signaling, inflammatory pathways are important both in the development of heart failure^24^,^25^,^26^ and in disease progression^41^. The JAK/STAT genes that are differentially expressed in RCM include *STAT3, STAT4*, interleukin receptors (*IL1RL1, IL18BP, IL4R, IL12RB1,* and *IL5RA*), and *JAK2* (Fig. 2B). In mouse studies, *STAT3* has been shown to be cardioprotective and anti-apoptotic in DCM^24^,^25^,^26^. Interestingly, in our RCM data set we see a 2.5-fold decrease in *STAT3* mRNA expression, accompanied by a 2.5-fold increase *JAK* mRNA expression (Fig. 2B). This is similar to reports on patients with DCM, in which protein levels of *STAT3* were reportedly decreased by ~50%^43^. Furthermore, it has been proposed that the decreased expression of *STAT3* in diseased hearts may contribute to the progression of heart failure^24^,^26^.

**Figure 7:**
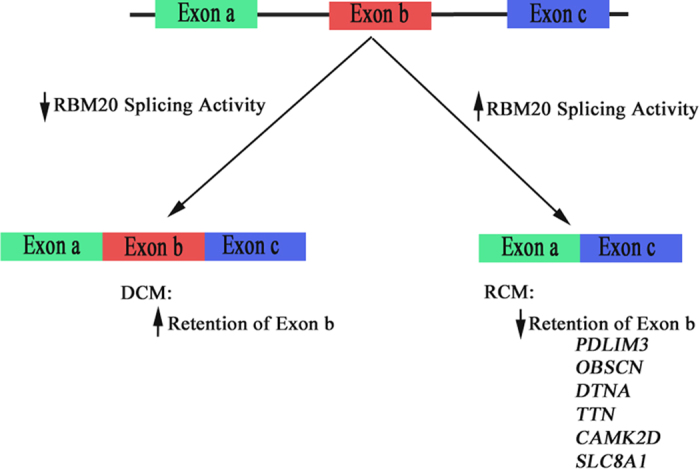
Model of RBM20 mediated splicing. A schematic is shown for a hypothetical gene, demonstrating exon inclusion when RBM20 mediated splicing activity is decreased, and exon exclusion when activity is increased.

There is a growing wealth of literature suggesting the importance of alternative splicing and splicing factor regulation in cardiomyopathy; however, the regulation of these splicing mechanisms in the pediatric RCM patient population has not been investigated. Here we present a detailed characterization of the alternative splicing regulation in RCM, where the alternatively spliced genes are associated with cardiomyopathies, ventricular outflow obstruction, regulation of heart contraction, actin cytoskeleton, and Z-disk. We identified genes with alternative splicing profiles that were common to cardiomyopathy (*PDLIM3, CD36, CLDND1, TTN, FAM126a, TLR4,* and *CD59*). Taken together, these results further support that alternative splicing regulation is a vital component of cardiomyopathy pathophysiology, including RCM. Interestingly, we found several genes to be mis-spliced in the opposite direction when comparing a DCM dataset with our RCM data. Significant differential splicing of cardiac contractile regulators were identified in RCM (*TTN* and *CAMK2D*), which are known targets of RBM20 (Figs 5 and 6). RBM20 is a well characterized splicing regulator that has been implicated in DCM progression^7^,^8^. Decreased levels of RBM20 in patients with DCM leads to retention of commonly spliced exons in several genes, including *TTN* and *CAM2DK*^7^,^8^. Other studies demonstrate that *TTN* is frequently mis-spliced in cardiomyopathy. We compared previously reported RBM20 predicted spliced targets in a dataset of high and low RBM20 expressing cardiomyopathies^7^,^8^. Surprisingly, this analysis found both *TTN* and *CAMK2D* to be mis-spliced in the inverse direction as our RCM dataset (Fig. 5). In summary, in DCM there is decreased RBM20 splicing resulting in the retention of commonly spliced exons (Fig. 7). However, in our model of RCM there is increased RBM20 splicing and subsequent decrease in retention of commonly spliced exons, including *PDLIM3, OBSCN, DTNA, TTN, SLC8A1*, and *CAMK2D* (Fig. 7). Taken together, these results suggest that the alternative splicing in RCM is potentially mediated by RBM20, however further studies are needed to verify the precise role of RBM20 in RCM disease progression.

Limitations to this study include the rarity of pediatric RCM subjects and the limited access to ventricular samples for both cases and controls. Furthermore, two of our RCM samples are from siblings (RCM1 and RCM2, FLNC) which decreases the genetic variability of our sample population. We have normalized each dataset to respective controls for that cohort to minimize platform-specific differences, but the data nevertheless represent merged datasets. Replicate studies with increased numbers of age, gender, and sample-site matched controls will be important.

In summary, we present the first transcriptional analysis of pediatric RCM and description of the molecular pathways that are dysregulated. These results are further compared to transcriptional analyses from adult DCM and heart failure, identifying both shared and divergent transcriptional dysregulation. In addition to gene regulation, we presented a detailed analysis of the abnormal alternative splicing patterns in pediatric RCM and identify RBM20 as a potential mediator of alternative splicing in this disease.

## Methods

### Sample collection and RNA purification

Myocardial samples were obtained from 4 patients with RCM and 5 controls. Subjects with RCM were diagnosed according to international guidelines^44^. Subjects with familial RCM who were listed for cardiac transplantation were ascertained from cardiomyopathy clinic. There were no history of myocarditis, infiltrative disease, or drug-induced cardiomyopathy.

Control experimental samples were collected by the National Disease Research Interchange (NDR) at the time of organ harvest from patients with a cause of death unrelated to cardiac failure (Table 1). Cardiac gene expression profiles are maintained for 24 hours in deceased subjects^45^. All NDR controls had normal left and right ventricular systolic function without regional wall motion abnormalities by echocardiography at the time of harvest. Total RNA was isolated from the cardiac samples utilizing the RNeasy Micro kit (Qiagen), according to the manufacturer’s recommendations. RNA quantity and quality was determined with the Agilent RNA 6000 Pico Chip (Agilent Technologies), yielding RNA concentrations of 5–10 ng/μl with RNA integrity numbers (RIN) greater than 9.0. This study was approved by the Institutional Review Board at Cincinnati Children’s Hospital Medical Center, Cincinnati Ohio, and carried out in accordance with regulatory guidelines and according to the ethical principles in the Declaration of Helsinki. All RCM subjects gave informed consent.

### Library Preparation and RNA-Seq

Due to low RNA concentrations resulting from the limited tissue obtained in pediatric myocardial biopsies, the Ovation RNA-Seq System v2 (NuGEN) was used to amplify the RNA and prepare cDNA according to the manufacturer’s protocol. The Nextera XT DNA Sample Preparation kit (Illumina Technologies) was utilized to generate the libraries. Briefly, 1 ng of cDNA was suspended in Tagment DNA Buffer followed by fragmentation and tagging with adaptors with the Nextera enzyme (Amplicon Tagment Mix). The samples were then neutralized with NT Buffer. Libraries were prepared by PCR with the Nextera PCR Master Mix, and 2 Nextera Indexes (N7XX and N5XX) according to the following program: one cycle of 72 °C for 3 minutes, one cycle of 98 °C for 30 seconds, 12 cycles of 95 °C for 10 seconds, 55 °C for 30 seconds, and 72 °C for 1 minute, and one cycle of 72 °C for 5 minutes. The purified cDNA was captured on an Illumina flow cell for cluster generation. The resulting cDNA library was sequenced utilizing the Illumina HiSeq 2500 platform (Illumina), at a targeted depth of 50 million paired-end, stranded 75 nucleotide reads.

### Data Analysis

The RNA-sequence FASTQ files were aligned to the human genome version hg19 and associated UCSC genome browser transcript GTF file using the software RSEM version 1.2.15^46^ for gene expression analyses and TopHat version 2.0.13^47^ for alternative isoform analyses. Differential gene expression was evaluated in AltAnalyze version 2.0.9^48^, using a moderated t-test p < 0.05, following a Benjami-Hochberg adjustment. Alternative splicing was also assessed in AltAnalyze using the reciprocal-isoform PSI method for both known and novel exon-exon junction^49^. This implementation of the PSI method is junction-centric and ignores sample measurements where insufficient depth is present (5 reads per junction in any junctions within the compared interval). Integrated heatmap, pathway, ontology and gene-set enrichment were performed with the software GO-Elite from AltAnalyze, in addition to PCA, Venn diagram and gene-network analyses^50^.

We integrated RNA-Sequencing data from existing adult datasets, available from the Gene Expression Omnibus (GEO, http://www.ncbi.nlm.nih.gov/geo/) repository^51^, utilizing the software AltAnalyze^48^ to identify crucial similarities and differences. To avoid the technical challenges of merging such datasets, given different sequencing approaches and processing protocols, each dataset has been normalized to the respective adult controls specific for that cohort, and subsequently used to generate a large common cardiomyopathy dataset combining our RCM dataset and the previously published ICM and DCM (GSE48166 and GSE55296) datasets. Given that such normalization produces large sets of regulated cardiomyopathy gene expression changes in the Common Cardiomyopathy dataset (n = 431), we believe such an approach is reasonable for defining common and disease-specific differences, including those underlying systolic versus diastolic dysfunction. Additionally, we generated RCM/ICM and RCM/DCM datasets in a similar manner, which combined our RCM dataset with either the previously published ICM (GSE48166) or the DCM (GSE55296) dataset, respectively.

## Additional Information

**How to cite this article:** Rindler, T. N. *et al*. Molecular Characterization of Pediatric Restrictive Cardiomyopathy from Integrative Genomics. *Sci. Rep.* **7**, 39276; doi: 10.1038/srep39276 (2017).

**Publisher's note:** Springer Nature remains neutral with regard to jurisdictional claims in published maps and institutional affiliations.

## Supplementary Material

Supplementary Information

## Figures and Tables

**Table 1:** Cardiac Tissue for RNA-sequencing.

Sample	Group	Source	Age	Sex	Mutation (RCM cases) or Cause of Death (Controls)
NDR1	Control	Explanted (organ harvest), left ventricle	67	F	Cerebral Vascular Accident
NDR2	Control	Explanted (organ harvest), left ventricle	29	F	Head Trauma
NDR3	Control	Explanted (organ harvest), left ventricle	57	F	Head Trauma
NDR4	Control	Explanted (organ harvest), left ventricle	37	F	Cerebral Vascular Accident
NDR5	Control	Explanted (organ harvest), left ventricle	26	M	Anoxia (Crush)
RCM1	RCM	Cardiac catheterization, right ventricle	7	F	*FLNC* (p.Pro2298Leu)
RCM2	RCM	Cardiac catheterization, right ventricle	11	M	*FLNC* (p.Pro2298Leu)
RCM3	RCM	Explanted (transplant), left ventricle	20	F	*TNNT2* (p.Glu163del)
RCM4	RCM	Explanted (transplant), left ventricle	10	F	Unknown

**Table 2:** Cardiac Features in RCM Subjects.

	RCM1	RCM2	RCM3	RCM4
*Echocardiography*				
LA Enlargement	**+**	**+**	**+**	**+**
Indexed LAV, ml/m^2	**72**	**62**	**45**	**74**
RA Enlargement	**+**	**+**	**+**	**+**
LV Dilation	–	–	–	–
LVEDD, mm (z-score)	3.7 (+0.2)	4.0 (+0.6)	5.1 (+0.3)	3.2 (−0.2)
LVH	-	-	-	-
IVS, mm (z-score)	0.6 (+0.1)	0.7 (+0.8)	0.8 (+0.1)	0.5 (−0.4)
PWT, mm (z-score)	0.7 (+1.0)	0.7 (+0.9)	0.7 (−0.3)	0.5 (−0.3)
Indexed LVM, g/m	36	32	32	34
LV Systolic Dysfx	–	–	–	–
LV FS, % (z-score)	35 (+0.1)	37 (+1.3)	35 (+1.0)	34 (−0.8)
LV Diastolic Dysfx	**+**	**+**	**+**	**+**
*Catheterization*				
LVEDP, mmHg	**18**	**17**	**14**	**20**
RVEDP, mmHg	**10**	**17**	**14**	**12**
Mean RAP, mmHg	**8**	**13**	**8**	**7**
PA Hypertension	–	**+**	–	**+**
Mean PAP, mmHg	22	**28**	20	**27**
PWP, mmHg	**16**	**20**	**15**	**16**
Rp, indexed Woods U	0.9	2.4	1.3	**4.5**
Cardiac Index, L/min/m2	5.4	4.6	3.8	4.9

Bold font indicates abnormal value; Dysfx, dysfunction (diastolic dysfunction as measured by patterns of mitral valve inflow); EDP end diastolic pressure; FS fractional shortening; LA left atrium; LAV left atrial volume (indexed to body surface area, normal <32 ml/m^2); LV left ventricle; LVM left ventricular mass (indexed to height ^2.7, normal <38-40 g/m); PA pulmonary artery; PAP pulmonary artery pressure; PWP pulmonary wedge pressure; RA right atrium; Rp pulmonary resistance.

**Table 3:** Summary of the Datasets and Comparisons.

Data Set/GEO Number	Description	Type	Source
NDR	Controls from NDR, n = 5	RNA-Seq	Experimentally Determined
RCM	RCM subjects, n = 4	RNA-Seq	Experimentally Determined
GSE48166	adult ICM (n = 15) and adult non-failing control hearts (n = 15)	RNA-Seq	Gene Expression Omnibus
GSE55296	ICM (n = 8) and DCM (n = 8) compared with non-failing control hearts (n = 8)	RNA-Seq	PMID: 24599027
GSE57338	non-failing left ventricle (ages 8-80yrs, n = 135)	HuGene-1_1-st	PMID: 25528681
GSE36761	left vs right ventricle	RNA-Seq	PMID: 24459294
**Combined Sets Names**	**Data Set**		
Non-failing	NDR and non-failing controls from GSE48166 and GSE55296, n = 28		
Cardiomyopathy	RCM and ICM (GSE48166 and GSE55296) and DCM (GSE55296), n = 35		
**Comparison Name**	**Description**	**Comparison**	
Common Cardiomyopathy	Comparison of all cardiomyopathy samples to non-failing hearts	Non-failing vs Cardiomyopathy	
NDR vs RCM	Comparison of RCM to control hearts	NDR vs RCM	
RCM specific	Comparison of RCM to common cardiomyopathy signatures	RCM vs Common Cardiomyopathy	
