# Supplementary material for: Molecular Characterization of Pediatric Restrictive Cardiomyopathy from Integrative Genomics
Source: Sci Rep. 2017 Jan 18;7:39276. doi: 10.1038/srep39276 (PMC5241776; doi:10.1038/srep39276)
Supplement: Supplementary Information [file srep39276-s1.doc]

Supplemental Data

Tara N. Rindler^1^, Robert B. Hinton^1^, Nathan Salomonis^2^, and Stephanie M. Ware^3^

Molecular Characterization of Pediatric Restrictive Cardiomyopathy from Integrative Genomics

**Supplemental Figure S1. Heat map clustering genes identifies in principal component 3 (PC3)**. PC3 ordered samples and genes (hierarchical clustering) segregated the samples according to disease status.


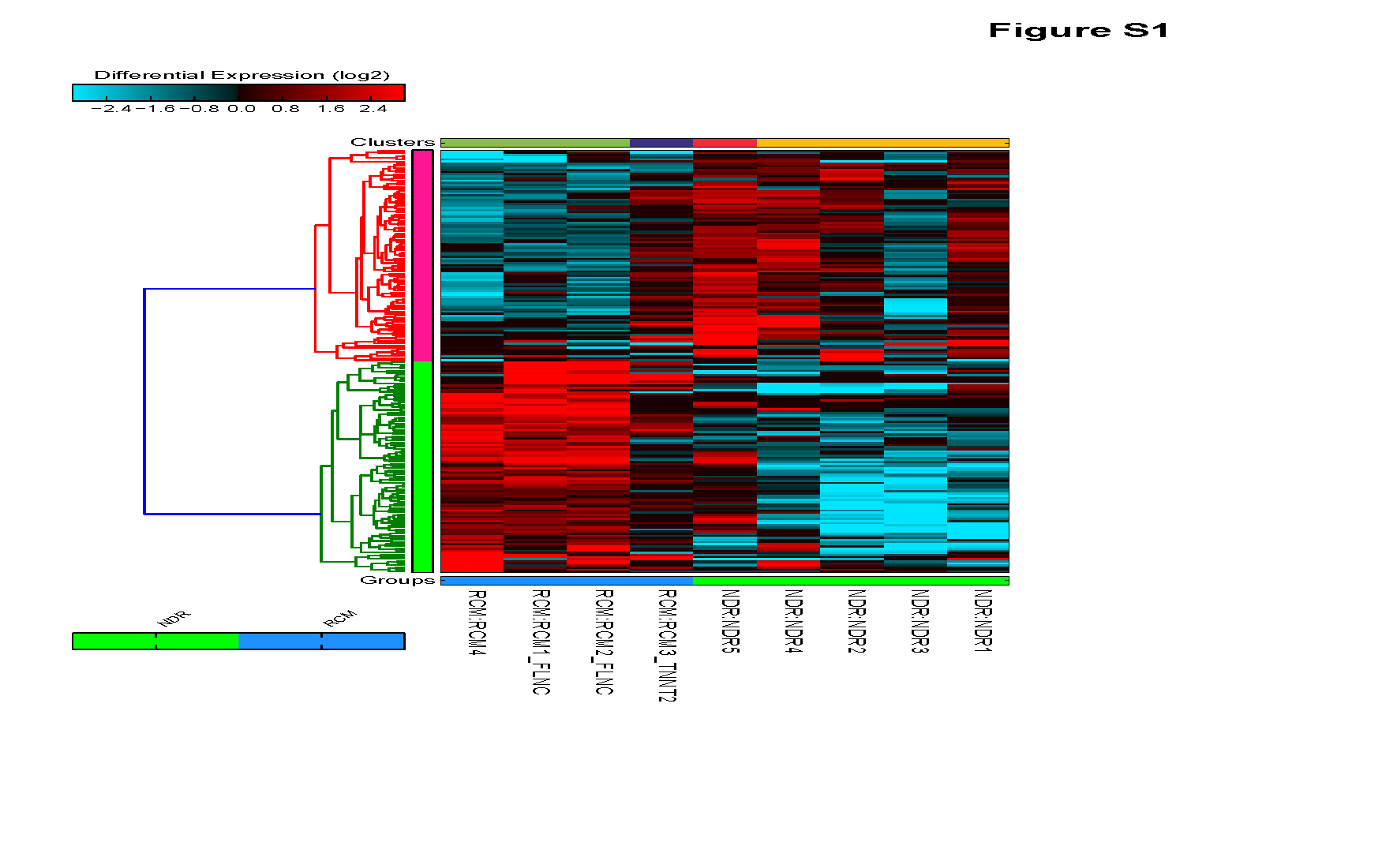


Supplemental Table 1

| RCM-increased | RCM-decreased | ICM-increased | ICM-decreased | DCM-increased | DCM-decreased |
| --- | --- | --- | --- | --- | --- |
| ITGAL | COL7A1 | SARDH | RP11-701P16.2 | NPPA | C1orf51 |
| DPT | SDK2 | IGFBP3 | LRRC30 | SMOC2 | ETS2 |
| DBP | MCM10 | COLEC11 | RARRES1 | SLC16A9 | JUNB |
| CNTF | XPNPEP2 | WT1 | GFRA4 | EGLN3 | TSPEAR |
| APLNR | NPTX2 | SLC25A33 | CXCL11 | HSPA2 | IL17RB |
| SEZ6L | MTFP1 | LHX6 | OR5B2 | TNFRSF12A | GBP4 |
| QPRT | ALPL | SSC5D | GKN1 | CILP | LRRC66 |
| CLIC6 | GPR4 | UQCR11 | PRR19 | ODC1 | ADRB1 |
| LRFN5 | LINC00327 | HES4 | RIPPLY2 | ENAM | ZNF331 |
| KIAA1199 | IL4R | RP11-10A14.4 | IDO1 | CLU | BATF2 |
| HLA-DPA1 | C3orf52 | COPS7B | KRT222 | ARNTL | PARP14 |
| SMOC2 | MPO | NDUFA7 | LCN10 | ANGPTL1 | CCL2 |
| GSDMC | FCGBP | BEST3 | GFRAL | LTBP2 | CSF1 |
| DLGAP1 | RRP12 | KDM8 | INSL3 | GLRB | FOSB |
| OIP5 | MAP3K6 | LOH12CR2 | ANKRD22 | MYL12A | IRF1 |
| ZNF582 | ZFYVE28 | MKI67 | RARRES3 | MLYCD | NLRC5 |
| HAPLN1 | DUS1L | KCNN3 | SERPINA3 | THBS4 | SREBF1 |
| ARHGAP28 | MARCO | FABP4 | PRAMEF2 | SPOCK2 | TSPYL2 |
| RSAD2 | SMPD2 | LFNG | KIR2DL4 | ST6GALNAC4 | VCAM1 |
| LOC400456 | LOC728175 | IFI27L2 | TNFSF14 | FAM150B | FAM167B |
| ABCG2 | LMNA | FGFR3 | OR52N4 | PLCE1 | ZNF195 |
| FCER1A | NACAD | ISLR | CXCL9 | ALDH4A1 | MYLK4 |
| CRYM | MAP1B | SPON2 | PCP4L1 | GPA33 | PRELID2 |
| TMEM163 | TAS2R30 | RPL37A | BATF2 | PTGDR2 | TAP1 |
| EVI2A | ZBTB7B | PRELP | CCL19 | MFAP5 | MPP3 |
| DKFZP686I15217 | PCNT | FAM227B | CSF3 | CENPA | PLA2G4F |
| DIO2 | PCDHGB2 | NUDT14 | CRISP2 | ASPN | RGS16 |
| CD200R1 | LOC100133920 | MEX3B | CXorf48 | UCHL1 | RARRES1 |
| CDK15 | CPNE5 | TUBB2A | TMEM61 | STAT4 | ICAM1 |
| FAM216B | ZDHHC12 | C9orf40 | CXCL10 | CCDC36 | COL27A1 |
| HLA-DRB5 | DUSP23 | SOX4 | SELE | SMAD7 | CEACAM19 |
| TNNT3 | HSD17B7P2 | PTRH1 | EDN2 | KAZALD1 | IFIT3 |
| GUCA1C | LGR6 | ELN | MAGEA4 | TPM3 | NR4A3 |
| PTK7 | APOB | C1orf220 | HAMP | PRELP | GNMT |
| LRRC14B | S100A8 | ABCG4 | CYP21A2 | FMOD | CDCA2 |
| MATN2 | S100A3 | RTN4R | GSDMA | JAK2 | ADAM11 |
| HBA1 | HOGA1 | CTF1 | CCL23 | KIF20A | GBP1 |
| ECT2L | TMEM14E | C3orf33 | PTX3 | MAMDC2 | CPLX3 |
| NMNAT2 | CPN2 | WDR54 | RND1 | KCNC3 | MT-ND1 |
| TYRP1 | ECE2 | C5AR1 | CFB | SCN2B | ATP1B3 |
| SLC44A5 | TFRC | APLNR | HLA-DOB | C8orf47 | SOCS3 |
| HBB | USP32P1 | NRARP | ALOX12B | DIRAS3 | MT-ND2 |
| A1BG-AS1 | SUSD4 | KAZALD1 | FRMD7 | CLSTN2 | PLA1A |
| CSNK1A1P1 | PLA2G2A | TYRO3 | FSHR | LPHN3 | BCL3 |
| EDIL3 | BCL6 | CNTN4 | ZNF195 | NUP62CL | SLC1A4 |
| LOC643529 | POR | SPOCK2 | TAS1R2 | SDSL | RAPGEFL1 |
| FBXO32 | LOC90834 | ACTA1 | C15orf38-AP3S2 | C14orf132 | RASD1 |
| DTX4 | SLC7A1 | GATM | KLK8 | KIAA1244 | NR4A2 |
| APBA1 | RIN1 | PTPRN2 | ETV7 | CNNM4 | PER3 |
| C1orf110 | GGT1 | MIF | PLA2G2A | MAPT | IDO1 |
| LOC100131096 | GGT5 | NME5 | GBP1 | ST8SIA2 | CHDH |
| PI16 | CHRDL2 | CDC42EP5 | LRRC66 | SEZ6L | MT-ATP8 |
| GPR162 | LOC646329 | NDUFC2 | MIOX | OMD | GBP2 |
| C5orf4 | PLP2 | COL1A2 | GPR88 | NAP1L3 | EPSTI1 |
| BMF | PCDHB13 | COCH | FGB | SFRP4 | SEMA4B |
| LRRC39 | NGFR | HAAO | RP11-712L6.5 | SHISA2 | IFI44L |
| ADCY10 | P2RY12 | CPXM1 | SPAM1 | GATM | SELE |
| CCDC36 | SLC25A18 | ASPN | SERPINE1 | SLITRK4 | TAP2 |
| OASL | LMCD1 | ARNTL | LINC00346 | LUM | APOL1 |
| CX3CR1 | TUBA3E | CNNM4 | TLCD2 | LRRC38 | FOS |
| ACE | TUBA3D | OXER1 | IRF1 | SPATA4 | PSME2 |
| MAGEA10 | STAT3 | SLC43A2 | IL17RB | PODN | MID1IP1 |
| MCOLN3 | ZFP91-CNTF | SH3TC2 | CD5L | FIBIN | GBP3 |
| PTPRT | FUK | GPR183 | OPRK1 | CNTN4 | LPCAT4 |
| PTPRR | ONECUT2 | ASF1B | NHLH1 | RP11-3B7.1 | MT-ATP6 |
| HHIP | CEACAM19 | SERINC2 | SLC16A8 | PDLIM3 | MT-ND4 |
| LOC100506368 | RAB15 | SDSL | SECTM1 | CTF1 | MT-CO2 |
| TTLL7 | CRLF1 | GADD45G | GNMT | HRK | IL18BP |
| SLC35F1 | FCN3 | SAMD14 | CIITA | DLK2 | SH3BP2 |
| TSPAN11 | GPR89C | SLC30A2 | BDKRB1 | FAM211A | ELL2 |
| KIAA1324L | FOSL1 | OGN | IL18BP | QPCT | PRMT10 |
| CLDN1 | METTL7B | CHAC2 | SLAMF1 | ACSM3 | RND1 |
| IRF6 | INO80B | NANOS1 | PRSS46 | GDF10 | APOL3 |
| LINC00282 | MYO1H | FCRLB | GDPD4 | SEZ6L2 | RCN1 |
| MIR100HG | CSPG4 | AMICA1 | RXFP2 | CA3 | BLM |
| TRIM65 | TMEM189 | CREB5 | GRXCR2 | PI16 | MT-CYB |
| ITK | SREBF1 | LMO3 | LILRA6 | C1QTNF9 | NR4A1 |
| CILP | COX6B2 | GPR179 | BPIFB3 | CRHBP | SHCBP1 |
| HLA-DQB2 | LRP1B | CSF2RA | C15orf48 | SLC16A6 | 11-Mar |
| KIT | C1orf170 | CD3D | AC003101.1 | CRLF1 | APOL2 |
| DHRS11 | GPR183 | EDIL3 | TLR3 | TTLL7 | FKBP1B |
| KCNE1 | MGST1 | KLF4 | FOSL1 | THY1 | ST6GAL2 |
| FNTB | KCNIP2 | CCNA2 | C4B | MCAM | ZFP36 |
| BCHE | SPP1 | IMMP1L | TAP1 | STK32A | SLCO5A1 |
| IGSF10 | AASS | SOX17 | PRSS45 | TIMP4 | EDN2 |
| C2orf40 | FKBP5 | KIF4A | TSSK3 | CCDC113 | ABCG1 |
| BACH2 | MFSD2A | PGPEP1L | CYP3A43 | BMP6 | CYP21A2 |
| KCNIP1 | LCN10 | NLRC3 | IFIT3 | CAPN6 | IFI16 |
| MYLK4 | KLC2 | LIME1 | AGXT2 | POSTN | PTX4 |
| GLP1R | GLYCTK | NPPC | TOX | CREB5 | MX2 |
| LINC00672 | PFN4 | NAP1L3 | DNAH3 | MME | CHGB |
| LOC550112 | MCTP2 | C4orf46 | SLC2A14 | STK17A | L3MBTL4 |
| BANCR | FKBP11 | PAFAH2 | OXGR1 | ARMS2 | PER1 |
| RABGAP1L | PNKP | RIMBP2 | SPIN2A | C3orf83 | PLEKHF1 |
| SHISA2 | AEN | IQGAP3 | ATP13A5 | CCL18 | KLKB1 |
| ACSM3 | TSPYL2 | SCN3B | CCL2 | ACTA1 | BTG2 |
| TMEM74 | CSDC2 | C1orf54 | GBP2 | MSS51 | PARP12 |
| ECM2 | C15orf59 | FSTL3 | GBP3 | FAM181B | PARP9 |
| LUM | BCL2L2-PABPN1 | C6orf25 | C3 | FAM86C1 | PSMB9 |
| TNNI1 | TMEM234 | DLK2 | L3MBTL4 | OGDHL | CCL11 |
| ABHD8 | GNMT | RP11-3B7.1 | SOCS3 | MXRA5 | CEBPD |
| FZD2 | ESCO2 | PROM1 | CHI3L1 | AEBP1 | WARS |
| BCL2L11 | CEP170B | TPM3 | GBP4 | NAALADL2 | GOLGA8B |
| FSBP | GFPT2 | COL1A1 | STAT1 | CPT2 | IFI44 |
| MME | ADAMTSL4 | C21orf7 | MUC1 | PROM1 | ODF3B |
| SLC24A3 | S1PR3 | SPNS3 | NKAIN4 | SLC43A2 | HSBP1L1 |
| STARD4-AS1 | FANCA | HSPA2 | ZNF331 | TMEM253 | ADCYAP1R1 |
| ITLN1 | SUN2 | POTEG | ADAMTSL1 | LIPE | PAX3 |
| SLC25A5-AS1 | TYMP | UQCRQ | FKBP1B | ZNF541 | TEF |
| C21orf91 | IQCJ-SCHIP1 | NETO2 | APOL6 | KCNE3 | FLT3LG |
| GADL1 | TOP3B | ZNF257 | CCL8 | C10orf67 | STEAP3 |
| HAS3 | ARPC4-TTLL3 | LCN12 | BTNL2 | NANOS1 | PLSCR1 |
| LOC283143 | MT2A | LEFTY2 | UBE2U | TSPAN8 | ZC3HAV1 |
| OGN | TIMP1 | C9orf96 | MAGEB17 | PENK | FGF18 |
| CTSK | KCNK1 | CPN2 | NLRC5 | AMY1A | ETV7 |
| CTSF | KCNK3 | RP11-111M22.2 | WDR49 | RP11-279O9.4 | DNAH3 |
| AFF3 | GOLGA8DP | GNAZ | ZNF486 | ATP1A1OS | LSAMP |
| THRSP | LOC729737 | MTRNR2L1 | C21orf128 | APCDD1L | MARVELD2 |
| FLRT2 | MT1X | HSPB1 | BCMO1 | HSPA8 | SLC2A5 |
| UBE2U | CCL11 | QPCT | TTYH1 | FAP | DARC |
| LFNG | FAM86FP | PKHD1 | RP11-766F14.2 | MYOC | MT3 |
| PENK | COL23A1 | DPEP2 | AC005082.1 | LNP1 | IL15 |
| BIRC7 | NEAT1 | RPH3AL | PLA1A | C21orf7 | RELN |
| RMI2 | MT1A | CXCR4 | CYSLTR1 | RUNDC3A | HLA-F |
| HPGDS | SLCO4A1 | POSTN | SLCO4A1 | ADAP1 | GRXCR2 |
| NFKBID | TMEM63C | RUFY4 | IFIT2 | LEPREL1 | SOCS1 |
| ANKRD34C | SGK110 | P2RX6 | MTFR2 | CRISPLD1 | CPNE5 |
| SLC9C1 | STAG3 | SSUH2 | CYP11B1 | SPON2 | C17orf53 |
| SLITRK4 | PNMT | C2orf40 | EPPIN | LPAR4 | LGALS9 |
| FRRS1L | AGAP4 | TMEM119 | FBXW12 | ZMYND12 | DDX58 |
| MSTN | LOC100134259 | PTK6 | CTD-2600O9.1 | NPPB | ATF3 |
| GZMA | ARHGEF39 | EGLN3 | BSPRY | ANTXR1 | FGF7 |
| RET | RHBDF2 | STK32A | CORO1A | SLC6A1 | GFRA1 |
| LEF1 | FBXO2 | AOAH | LCN6 | GPR173 | MOV10 |
| CA3 | ZMIZ1-AS1 | CLIC3 | SLCO5A1 | NDNF | SCD |
| APCDD1L | MIDN | MT1E | KEL | SPNS3 | CCDC85A |
| SLAMF7 | PCBP3 | TMEM139 | PTGES | SMARCE1 | PTGES |
| TGFB2 | HRCT1 | FOXS1 | MYLK4 | OGN | IL3RA |
| FCRL3 | PAPPA2 | PCDH19 | TAS2R10 | PDE8B | DMKN |
| FCRL6 | C1QTNF1 | MYL12A | PAX3 | SSTR5 | C4B |
| RUNX2 | PVRL1 | S100B | CDCA2 | SCRG1 | UBE2L6 |
| RUNX3 | SLC17A9 | PDE4C | GPR84 | TTC9 | RAB3IL1 |
| GRIA1 | C19orf59 | COL3A1 | ITGAD | ESM1 | RP11-1105G2.3 |
| SCUBE2 | AQP3 | BIRC5 | OASL | DUSP10 | FAAH |
| ESR1 | GNB1L | CRHBP | IL15 | ZCCHC5 | IFIT2 |
| FAP | ITPK1 | CAPN6 | ELF3 | KCNN3 | VASH2 |
| NPAS3 | FANCD2 | MYL7 | PARP9 | CHST9 | IFI35 |
| C7orf41 | BAI2 | ZCCHC5 | CEBPD | SCN3A | LRRC4C |
| PDE7B | GADD45B | TNNT3 | FGF18 | IQCD | CSF3 |
| GPR22 | MMP23A | ZNF816 | ABCG1 | ADM | PNMA3 |
| AMT | FAM35DP | NAALADL2 | PARP14 | CTD-2267D19.3 | CCDC73 |
| RASL11B | SH3BGRL3 | NCAPH | ELL2 | TRIM50 | SLC36A2 |
| EPHA3 | LINC00085 | HAPLN1 | LILRA5 | FNDC1 | MYH6 |
| COL9A1 | GALNT15 | CDKN1C | USH1C | CTHRC1 | ASB12 |
| FLJ39739 | LRP8 | UBE2C | FCGR3A | MT1G | CORIN |
| C1QTNF9 | PPL | ACSM3 | C10orf10 | GLP1R | IRF9 |
| C1QTNF7 | CRYM-AS1 | THBS4 | OSMR | GPR128 | GPR35 |
| SLC6A1 | LOC284837 | RGS1 | KLF5 | FSTL3 | SLAMF1 |
| AMICA1 | RNF103-CHMP3 | F2 | CHGB | KCNA6 | PZP |
| C3orf43 | LRRC19 | FAM86C1 | ZNF286B | FREM1 | HAPLN3 |
| FLJ42969 | AQP7P3 | ADM | APOL1 | NR2F1 | MYO1H |
| SLC4A1 | AQP7P1 | LPAR4 | GZMB | MIXL1 | ANGPT2 |
| ASPN | NCAM1 | CHST9 | HTR2B | APLP1 | PRR22 |
| MKX | CCDC107 | CTHRC1 | PRF1 | ZC3HAV1L | ACR |
| IGSF6 | CD163 | GATA3 | SPEF2 | CYP11A1 | GABBR2 |
| MYOC | CPAMD8 | MXRA5 | EPSTI1 | GPR1 | SERPINE1 |
| TMEM255A | RCC1 | OGDHL | RANBP3L | SLC25A34 | SSTR2 |
| ATP1B4 | SIGLEC9 | FNDC1 | DTX3L | CTD-3088G3.8 | PSMB8 |
| ATP1B2 | FLJ42393 | TMPRSS13 | CALCB | CHODL | CASKIN1 |
| TESK2 | CEBPB | NRIP3 | IL1RN | ACADL | PP13439 |
| SIGLEC17P | AP3D1 | ADAM12 | GBP5 | MAPK4 | MTRNR2L3 |
| PIWIL4 | TMEM52 | DSG1 | SHCBP1 | NTRK2 | TOX |
| C6orf25 | EPHX3 | SMOC2 | KCNJ5 | NAP1L2 | ABCA7 |
| HLA-DPB1 | LRRC32 | RGS4 | C1R | SPINK1 | PRR26 |
| MMP23B | LY6G5B | ANKRD23 | PLA2G4F | ERICH2 | KYNU |
| OR2A9P | SYCP3 | NUSAP1 | DNAH10OS | SLPI | PCP4L1 |
| SULT1C4 | BMP8B | RP11-383H13.1 | SH2B2 | DEFB124 | CDKL4 |
| LOC100506469 | PTPN5 | C2CD4C | UCN2 | GSC | IL15RA |
| FNDC1 | CP | EME1 | KLK14 | PCDH10 | SLC22A4 |
| IDI2-AS1 | FLJ23867 | TESPA1 | SYT15 | GPR68 | SECTM1 |
| CD8A | GPR135 | RRM2 | DMRTA2 | ECT2L | C11orf52 |
| HLA-DOA | ALS2CL | KCNE3 | TMEM151B | ABP1 | ISG20 |
| ANTXR1 | TUBB4B | MMP24 | LYPD5 | LRRC43 | PTF1A |
| S100B | ADARB2 | MYEOV | PCDHGA11 | ZNF385C | RP11-758M4.1 |
| HLA-DQA1 | SND1-IT1 | CILP | TAP2 | SLC1A7 | DRAXIN |
| HLA-DQA2 | HOPX | GPR17 | ESPNL | MN1 | FRMD7 |
| KLRB1 | CHP2 | HBA2 | RRM1 | NT5C1A | FSHR |
| ASB14 | LMO3 | TNFRSF12A | EXPH5 | MYCN | PMP2 |
| PIK3R6 | CCDC73 | APLN | C15orf60 | KCNJ3 | C2CD4B |
| SORL1 | DDX11 | PARPBP | KCNK7 | MS4A4E | CIITA |
| CECR6 | SLX1A | TM6SF2 | TBC1D29 | SCAND3 | CXCL14 |
| LEPREL1 | SLX1B | KIFC1 | ZNF852 | USP6 | OPRK1 |
| DMKN | UBTD1 | SLC25A34 | MARCO | PDCD1 | SLFN13 |
| PRND | RRBP1 | PON1 | CD300E | LY9 | CAMSAP3 |
| CDCA7 | PPP1R14B | SFRP4 | BET3L | RP11-274K13.2 | LILRA1 |
| HLA-DMA | CHURC1-FNTB | TMEM163 | SLC5A10 | KERA | CHRNA6 |
| SYTL5 | COL17A1 | PRRT3 | PLEKHA7 | SAMD11 | DTX3L |
| ESRP1 | FOS | HR | CCL11 | ZNF681 | EGR3 |
| RELN | VSTM2L | SEZ6L2 | TUBA3E | NTRK1 | CA14 |
| CRISPLD1 | MROH7 | PTGDR2 | CDCP1 | PADI4 | SLC13A4 |
| IGF1 | SH2D5 | FAM129C | POF1B | TUBB2B | LYPD6 |
| FBXL22 | MAPK4 | ZNF541 | FAM115C | LMO3 | CXCL11 |
| LRRC3B | SERPINA3 | TCP10L | KCNQ3 | HAPLN1 | CTB-134H23.2 |
| TRIL | SLC38A10 | CCDC11 | RELN | PGPEP1L | MUC1 |
| NPY1R | TLCD2 | TRAM1L1 | KLHL40 | KRTAP12-3 | TNFSF14 |
| C4orf29 | GRB14 | SEZ6L | IFI30 | GPR179 | PNP |
| EGR2 | IL18BP | ALOX15B | ATP1B3 | NUSAP1 | CCL19 |
| ABCA9 | TOB2P1 | RAD21-AS1 | IFI44L | PRG4 | UBE2U |
| PRKAB2 | AKAP3 | KRT76 | PRMT10 | CLIC3 | NOD2 |
| GTF2A1L | PCDHGA4 | MYLPF | FKBP1C | PLEKHH2 | AQP3 |
| SFRP1 | ITPK1-AS1 | IL1RL2 | CCR1 | RLN2 | APOL6 |
| C9orf131 | CBS | TFF3 | ARG2 | CPN2 | SLA2 |
| KIAA1210 | TLCD1 | NUGGC | APOL4 | CNTFR | IRX2 |
| C11orf21 | MYC | MS4A1 | FCGBP | FRZB | SLC6A9 |
| COL6A6 | ENTPD6 | PALM3 | PRELID2 | IL17D | SLC6A20 |
| KLK13 | ASPG | C9orf169 | APOL2 | GNAZ | PLEKHA7 |
| BCDIN3D-AS1 | VDR | C1orf158 | OSCAR | MMP24 | C4A |
| BLNK | OSGIN1 | C4orf19 | UBE2L6 | DAZL | IL6 |
| PTN | MVP | POMC | CHRNA6 | TRAM1L1 | ZMAT4 |
| AMPH | GATA2 | NUP62CL | HUNK | COL9A1 | STAT1 |
| ACAN | LOC150776 | RUNDC3A | RP11-259P6.1 | C14orf144 | TYMP |
| UCP2 | SERPINE1 | PBK | VCAM1 | ZNF214 | MAGEA4 |
| HBA2 | KLHL25 | SAMD11 | PCDHGB3 | ACOT4 | ZDHHC19 |
| MSS51 | BEST3 | CTD-2260A17.2 | MYH6 | SARDH | BMPR1B |
| PDZRN4 | IL1RL1 | C8orf47 | PRG2 | GOLGA8R | BTN3A1 |
| EMILIN3 | PKP1 | ENHO | ACR | TMEM119 | KRT222 |
| LRRC55 | PKHD1 | OLFM3 | LRRC14B | CD5 | PRG2 |
| DENND1C | CRELD2 | SHISA2 | ZC3HAV1 | LAMP3 | ATP13A5 |
| SEPT5-GP1BB | SLC29A2 | KANK4 | CAMSAP3 | NRIP3 | TEX12 |
| FBXO17 | FLJ38109 | ZNF385C | SEMA4G | F2RL2 | TSPAN11 |
| CNGB3 | SPATC1L | RP11-274K13.2 | HDC | ENTPD3 | USH1C |
| GJB2 | HIF1A-AS2 | STAT4 | BOP1 | SCUBE2 | KIAA2022 |
| FOXI2 | SLC25A22 | RP11-597K23.2 | RASD1 | EMILIN3 | BCMO1 |
| BARD1 | MIIP | MATN3 | KLHL38 | FCRLA | BTBD16 |
| PM20D1 | RPUSD1 | LRRC55 | MUC20 | SFRP5 | TRIM22 |
| LOC148145 | GCSAM | OIT3 | IRF9 | FFAR4 | GNLY |
| LRRTM4 | RALYL | DCAF12L1 | C17orf53 | SLX4IP | IFIH1 |
| FREM1 | C1orf162 | HRK | ANKRD18B | PLCH1 | HUNK |
| SEC14L5 | PI15 | AKR7L | ENPP6 | FAM171A2 | HCN4 |
| GPR83 | AP1G2 | IL23A | C12orf61 | COCH | ANKRD66 |
| ADAM22 | FASN | ASCL2 | IFITM1 | ULBP2 | ADAMTS4 |
| GSTM5 | ZBTB42 | RNF183 | F11 | MCHR1 | SEC14L5 |
| LOC728463 | MFSD12 | WISP1 | C11orf52 | RSPH6A | KCNIP2 |
| GDF6 | TP53I13 | KCNA5 | PCDHGC4 | DMC1 | CD19 |
| ANKHD1-EIF4EBP3 | LINC00485 | GZMK | DTHD1 | GSG1L | AHNAK2 |
| ISLR | FAM20C | COL9A1 | NR4A2 | IL18 | MUC20 |
| MST1R | RDH5 | GREM1 | RASSF6 | HIST1H2AG | KIAA1257 |
| C9orf170 | NAP1L2 | NR2F1 | LRRC25 | RTN4R | ALPK1 |
| SLC25A45 | FAM58A | FAM163A | TNFSF10 | C1orf194 | CDC25A |
| TMCC3 | FLJ42875 | CETN1 | SLCO2A1 | TMPRSS11E | BATF3 |
| LOC374443 | POM121L9P | LY9 | HSPA6 | ANGPTL7 | CYP11B1 |
| CABLES1 | LOC401324 | SLC16A9 | AL592284.1 | TNNI1 | KIAA1456 |
| ROPN1 | SDF2L1 | CH25H | NKX2-3 | HIST1H2AK | BSPRY |
| RNF165 | AZGP1 | C3orf83 | CSF1 | ZNF788 | C8G |
| THY1 | BLM | FAM171A2 | MID1IP1 | LCN15 | FAM124A |
| ANGPTL7 | MMP19 | ANGPTL4 | NAMPTL | SLC5A4 | ERAP2 |
| MMP16 | SDCBP2 | SLC9C2 |  | DPEP2 | SLC2A14 |
| IL5RA | VASP | NEK2 |  | MADCAM1 | BATF |
| DLK1 | FAM46B | KLHDC7B |  | CTD-3148I10.1 | DMRTA2 |
| IFI44L | FBP1 | THY1 |  | SDC1 | SYT2 |
| RERGL | MAFF | MEP1A |  | LEFTY2 | IL8 |
| DPY19L1P1 | WISP2 | IGSF1 |  | SLC9C2 | CYP3A43 |
| PROM1 | KLHL40 | EPHA6 |  | CLEC10A | CES3 |
| SCARB1 | COMMD3-BMI1 | CDH8 |  | LYPD1 | POF1B |
| GATM | TMPRSS5 | GYPE |  | ASPHD1 | NSG1 |
| FAM129A | LOC100288778 | SH2D3A |  | PDE4C | AC016722.1 |
| BUB1 | HIP1R | SYN2 |  | FAM180B | TM4SF18 |
| DNAH5 | MPP3 | DNAH7 |  | MAP1LC3C | RP11-701P16.2 |
| CSF1R | SNX29P2 | TMEM253 |  | ITGBL1 | CXCL3 |
| CDKL2 | RGMA | LYPD2 |  | BBS1 | BTNL2 |
| CRHR2 | PRODH | IL1RAPL2 |  | PDE3B | ELF3 |
| IQGAP3 | BATF3 | SOHLH2 |  | WNT10B | NSG2 |
| SFRP4 | RSPH4A | PTTG2 |  | CHRNA5 | CHI3L1 |
| ALDH1A2 | GPRC5A | MBLAC2 |  | UNCX | PMFBP1 |
| ATP6V1G2 | NOMO3 | FAM181B |  | UNC80 | HDC |
| LOC145474 | CYP4B1 | CCDC65 |  | UCMA | APOBEC4 |
| CASP6 | LCN6 | OR7D2 |  | ALOX15B | C1orf148 |
| PKD1L2 | ADA | TNMD |  | AC021218.2 | CXorf58 |
| LINC00847 | GALK1 | FBXO43 |  | TAS2R19 | CD14 |
| PPP2R2B | RPL23AP32 | FCRLA |  | CABP7 | GBX2 |
| SEPT7P2 | AOX1 | C1QTNF9 |  | ALAS2 | GDF7 |
| HAAO | RNASE2 | SH3GL3 |  | NTN5 | CDHR4 |
| POLN | GPR56 | CIDEA |  | DSG1 | SLC13A3 |
| C2orf16 | DGKZ | TBR1 |  | DEFB1 | RSAD2 |
| DCX | AHNAK2 | ADIPOQ |  | HYAL4 | ADAMTSL1 |
| CHRDL1 | SEMA4B | CHODL |  | TAC4 | GBX1 |
| NREP | QSOX1 | MT1G |  | C5AR1 | KLHL40 |
| BAALC | EMC9 | RP11-279O9.4 | | GREM1 | CDC7 |
| RTN1 | ZSCAN5A | CENPA |  | ZNF536 | OSMR |
| PLXDC1 | TEAD4 | PCSK1N |  | ADAM20 | TMEM151B |
| ULK4P3 | LRRC9 | KIF20A |  | CIDEA | HOXD3 |
| GALNT8 | C5AR1 | CCDC33 |  | C22orf26 | OR2W1 |
| CD72 | ADAMTS4 | DEFB124 |  | IL1RL2 | CTD-2600O9.1 |
| NLRP9 | GMNC | NPPA |  | PPP1R42 | APOL4 |
| KIF1A | DHX37 | LGALS4 |  | ADIPOQ | GDF5OS |
| METTL21EP | MYH3 | PCDH10 |  | SLC24A4 | GAGE10 |
| MBP | MYH6 | ESM1 |  |  | HGFAC |
| LOC283731 | MYH9 | SDC1 |  |  | WDR49 |
| ZNF665 | PUS1 | ADCY8 |  |  | DBP |
| L3MBTL4 | FGD2 | C14orf144 |  |  | TBC1D29 |
| GFRA3 | SMN2 | NPPB |  |  | IL20RA |
| CPA3 | ATAD3B | FAM150B |  |  | IL27 |
| XKR4 | RIPK3 | TUBB2B |  |  | AL445665.1 |
| ANKRD33B | AQP4 | MYO6 |  |  | CCL8 |
| DOK6 | LOC100862671 | RIPK4 |  |  | KCNH8 |
| CD163L1 | BSPRY | CIDEC |  |  | SLC26A9 |
| C1orf204 | SLCO2A1 | LAIR2 |  |  | LIF |
| ARMC4 | OSMR | CACNG3 |  |  | FOXJ1 |
| GDNF | GPAT2 | NODAL |  |  | ZNF286B |
| CD226 | LOC100506195 | ATP12A |  |  | CCL23 |
| ADIPOQ | EHF | CLCN1 |  |  | ASAH2B |
| NET1 | MVK | AC021218.2 |  |  | DRAM1 |
| PDE5A | MGC16142 | NLRP8 |  |  | NNMT |
| C10orf25 | CES4A | PRR23A |  |  | NTNG1 |
| SESN3 | SIGLEC16 | BPIFA2 |  |  | MCTP2 |
| CTSW | EFNB1 | SLC34A1 |  |  | ZSCAN4 |
| GVINP1 | RAD51 | CLDN19 |  |  | GPC5 |
| LYPD6 | SYN2 | OR52E4 |  |  | TRIM21 |
| AMHR2 | CHRNB2 | RRH |  |  | C17orf72 |
| HMGCLL1 | SLC11A1 | SLC7A4 |  |  | HLA-DOA |
| ITIH5 | MAP3K10 | FFAR4 |  |  | SPDEF |
| PIEZO2 | RAP1GAP | SIGLECL1 |  |  | C15orf60 |
| GPR111 | DNAH14 | GPR128 |  |  | TTC6 |
| ISLR2 | ADAMTS15 | MS4A4E |  |  | PRRG4 |
| TET1 |  | CBLC |  |  | ATP2A1 |
| ST6GAL2 |  | DEFB1 |  |  | FAM115C |
| HSPE1-MOB4 | | CABP7 |  |  | GRIA4 |
| FMO1 |  | RLN1 |  |  | SYT8 |
| PLCE1 |  | PADI4 |  |  | SPTLC3 |
| DDIT4L |  |  |  |  | SPC24 |
| CD22 |  |  |  |  | CYP4V2 |
| LOC642846 |  |  |  |  | TUBB4A |
| FXYD6 |  |  |  |  | SLC17A9 |
| LPHN3 |  |  |  |  | ATP6V1B1 |
| HR |  |  |  |  | ADH1B |
| CRIP3 |  |  |  |  | MCTP1 |
| ANKRD23 |  |  |  |  | NEUROG3 |
| KIAA1211 |  |  |  |  | PHEX |
| PBK |  |  |  |  | GPAT2 |
| P2RY13 |  |  |  |  | DLGAP5 |
| PROX2 |  |  |  |  | CADM3 |
| TM7SF2 |  |  |  |  | UCN2 |
| NPR3 |  |  |  |  | CXCL10 |
| KAZALD1 |  |  |  |  | CCDC19 |
| LPAR4 |  |  |  |  | PCP2 |
| CD1C |  |  |  |  | CYP27B1 |
| JAK2 |  |  |  |  | C9orf171 |
| GRM1 |  |  |  |  | IL1F10 |
| TLL2 |  |  |  |  | HLA-DOB |
| GRM8 |  |  |  |  | S100A3 |
| RIMS1 |  |  |  |  | C3 |
| GRID1 |  |  |  |  | BET3L |
| APOBEC3D |  |  |  |  | CLNK |
| EXTL1 |  |  |  |  | TAS2R10 |
| KLRK1 |  |  |  |  | MIOX |
| JAM2 |  |  |  |  | RNASE13 |
| LOC100128682 | |  |  |  | PLAC1 |
| RGS4 |  |  |  |  | PRAMEF2 |
| SNCA |  |  |  |  | KCNMB3 |
| RCOR2 |  |  |  |  | OR4M2 |
| SLC1A2 |  |  |  |  | TVP23A |
| PODN |  |  |  |  | LINGO2 |
| CNIH3 |  |  |  |  | ELANE |
| METTL21B |  |  |  |  | ALOX12B |
| FMOD |  |  |  |  | EXPH5 |
| FRZB |  |  |  |  | MMP25 |
| MAL2 |  |  |  |  | AADACL3 |
| ALS2CR11 |  |  |  |  | PCSK1 |
| LOC100129480 | |  |  |  | C1orf191 |
| CCDC89 |  |  |  |  | B3GNT7 |
| LOC729950 |  |  |  |  | HPDL |
| STAT4 |  |  |  |  | MOCOS |
| OR51E1 |  |  |  |  | UNC93A |
| GPR75-ASB3 |  |  |  |  | PCDH11Y |
| DGKB |  |  |  |  | ZPLD1 |
| ICK |  |  |  |  | C21orf90 |
| ZNF169 |  |  |  |  | LINC00346 |
| ELOVL2-AS1 |  |  |  |  | NKD2 |
| LRP2 |  |  |  |  | IFI30 |
| FFAR4 |  |  |  |  | TDRD5 |
| EDARADD |  |  |  |  | CCL21 |
| MFSD4 |  |  |  |  | C10orf10 |
| PTGER3 |  |  |  |  | LYPD5 |
| THBS4 |  |  |  |  | CD80 |
| PGAM1P5 |  |  |  |  | EGFL8 |
| HYMAI |  |  |  |  | DDX11 |
| CENPA |  |  |  |  | DUSP9 |
| ACSM5 |  |  |  |  | SLC19A3 |
| VNN1 |  |  |  |  | WSCD2 |
| CD1E |  |  |  |  | KLHL32 |
| VGLL2 |  |  |  |  | BTC |
| NKG7 |  |  |  |  | GNG4 |
| COL24A1 |  |  |  |  | GLDC |
| SLC6A9 |  |  |  |  | FGF10 |
| VAT1L |  |  |  |  | SLC8A3 |
| FGF23 |  |  |  |  | KRTAP5-9 |
| ZCCHC5 |  |  |  |  | TMEM31 |
| CAPN6 |  |  |  |  | IL1RN |
| HBG2 |  |  |  |  | AP000783.1 |
| FATE1 |  |  |  |  | DRD4 |
| DCHS1 |  |  |  |  | GRIK4 |
| TNF |  |  |  |  | AC006946.15 |
| NAPSB |  |  |  |  | TDGF1 |
| NCR3LG1 |  |  |  |  | CYP1A1 |
| PLXNC1 |  |  |  |  | SCGN |
| PRDM6 |  |  |  |  | CYP2C8 |
| PRDM1 |  |  |  |  | C21ORF116 |
| ALK |  |  |  |  | MIA |
| PABPC4L |  |  |  |  | WNT10A |
| SCN8A |  |  |  |  | TACR3 |
| DKK2 |  |  |  |  | RP11-1070N10.3 |
| AVPR1A |  |  |  |  | NYAP2 |
| CERKL |  |  |  |  | CXCL1 |
| TCEANC |  |  |  |  | CPN1 |
| YPEL2 |  |  |  |  | CFTR |
| DPEP2 |  |  |  |  | CAPN11 |
| EFNB3 |  |  |  |  | ACPT |
| NDC80 |  |  |  |  | DMRTB1 |
| KIAA1549L |  |  |  |  | PLEKHS1 |
| TNFSF4 |  |  |  |  | SPEF2 |
| ESM1 |  |  |  |  | EMR1 |
| SOX4 |  |  |  |  | HOXB-AS3 |
| SOX5 |  |  |  |  | PDILT |
| FAM181B |  |  |  |  | TREX2 |
| INHBA |  |  |  |  | LGALS12 |
| PHKG1 |  |  |  |  | CRISP1 |
| CRHBP |  |  |  |  | NLRP13 |
| PIK3IP1 |  |  |  |  | KIF2B |
| PRTFDC1 |  |  |  |  | C9orf135 |
| CD5 |  |  |  |  | ARL17B |
| GABRB2 |  |  |  |  | CASC1 |
| SPATA6L |  |  |  |  | FAM22F |
| CCDC62 |  |  |  |  | CXorf67 |
| HBG1 |  |  |  |  | KLHDC8A |
| KCNA5 |  |  |  |  | BMP7 |
| BTBD8 |  |  |  |  | ENPP7 |
|  |  |  |  |  | AKR7A3 |
|  |  |  |  |  | TNFRSF13B |
|  |  |  |  |  | C10orf129 |
|  |  |  |  |  | HEPACAM |
|  |  |  |  |  | OXGR1 |
|  |  |  |  |  | PSD2 |
|  |  |  |  |  | FOXH1 |
|  |  |  |  |  | ANKFN1 |
|  |  |  |  |  | MYRFL |
|  |  |  |  |  | SLC5A10 |
|  |  |  |  |  | LIPJ |
|  |  |  |  |  | C15orf62 |
|  |  |  |  |  | ARID3C |
|  |  |  |  |  | BPIFB3 |
|  |  |  |  |  | CLEC4D |
|  |  |  |  |  | FCGBP |
|  |  |  |  |  | LRRC30 |
|  |  |  |  |  | AL049840.1 |
|  |  |  |  |  | GALNT9 |
|  |  |  |  |  | PF4 |
|  |  |  |  |  | DDX43 |
|  |  |  |  |  | TRIM40 |
|  |  |  |  |  | HMX1 |
|  |  |  |  |  | FCRL2 |
|  |  |  |  |  | CHP2 |
|  |  |  |  |  | PRR19 |
|  |  |  |  |  | MROH2B |
|  |  |  |  |  | RIPPLY2 |
|  |  |  |  |  | GPR88 |
|  |  |  |  |  | SLC17A1 |
|  |  |  |  |  | KRT5 |
|  |  |  |  |  | PRDM13 |
|  |  |  |  |  | RPS6KA6 |
|  |  |  |  |  | OR51J1 |
|  |  |  |  |  | TCTEX1D1 |
|  |  |  |  |  | ABCA4 |
|  |  |  |  |  | BCAS1 |
|  |  |  |  |  | NKX2-3 |
|  |  |  |  |  | TIMD4 |
|  |  |  |  |  | SYCP1 |
|  |  |  |  |  | FLRT1 |
|  |  |  |  |  | ITIH3 |
|  |  |  |  |  | ZAR1L |
|  |  |  |  |  | CAGE1 |
|  |  |  |  |  | TUBA3E |
|  |  |  |  |  | RARRES3 |
|  |  |  |  |  | GAS2L3 |
|  |  |  |  |  | CACNG8 |
|  |  |  |  |  | CHRM5 |
|  |  |  |  |  | ZNF503-AS2 |
|  |  |  |  |  | AGXT2 |
|  |  |  |  |  | SLC6A15 |
|  |  |  |  |  | MGAT4C |
|  |  |  |  |  | MC2R |
|  |  |  |  |  | HAL |
|  |  |  |  |  | USP43 |
|  |  |  |  |  | GJD3 |
|  |  |  |  |  | C1R |
|  |  |  |  |  | CLVS1 |
|  |  |  |  |  | SMLR1 |
|  |  |  |  |  | C10orf105 |
|  |  |  |  |  | PIPOX |
|  |  |  |  |  | SCGB1D2 |
|  |  |  |  |  | IRGC |
|  |  |  |  |  | RXFP2 |
|  |  |  |  |  | MUC19 |
|  |  |  |  |  | GUCY2C |
|  |  |  |  |  | DCC |
|  |  |  |  |  | RP11-830F9.6 |
|  |  |  |  |  | IL19 |
|  |  |  |  |  | CDHR2 |
|  |  |  |  |  | FAM71C |
|  |  |  |  |  | SLITRK5 |
|  |  |  |  |  | KLRC1 |
|  |  |  |  |  | SLCO1A2 |
|  |  |  |  |  | OR13C8 |
|  |  |  |  |  | CLDN20 |
|  |  |  |  |  | GDF3 |
|  |  |  |  |  | FFAR2 |
|  |  |  |  |  | OR4A15 |
|  |  |  |  |  | C22orf24 |
|  |  |  |  |  | GOLGA8F |
|  |  |  |  |  | TLCD2 |
|  |  |  |  |  | TMEM132E |
|  |  |  |  |  | SPATA16 |
|  |  |  |  |  | PRSS54 |
|  |  |  |  |  | THSD7A |
|  |  |  |  |  | ASMT |
|  |  |  |  |  | CD5L |
|  |  |  |  |  | CARD9 |
|  |  |  |  |  | FIBCD1 |
|  |  |  |  |  | MTFR2 |
|  |  |  |  |  | APOA4 |
|  |  |  |  |  | AC015987.2 |
|  |  |  |  |  | SAGE1 |
|  |  |  |  |  | REM2 |
|  |  |  |  |  | WNT6 |
|  |  |  |  |  | RTP1 |
|  |  |  |  |  | TRIM15 |
|  |  |  |  |  | AL162389.1 |
|  |  |  |  |  | SULT1C2 |
|  |  |  |  |  | ORC1 |
|  |  |  |  |  | GCM2 |
|  |  |  |  |  | RNF113B |
|  |  |  |  |  | PLA2G2F |
|  |  |  |  |  | SFTPB |
|  |  |  |  |  | PLEKHD1 |
|  |  |  |  |  | H1FOO |
|  |  |  |  |  | OR2AK2 |
|  |  |  |  |  | TMEM92 |
|  |  |  |  |  | ZNF804A |
|  |  |  |  |  | ARTN |
|  |  |  |  |  | CCL4L2 |
|  |  |  |  |  | GPR156 |
|  |  |  |  |  | EGR4 |
|  |  |  |  |  | RDM1 |
|  |  |  |  |  | PPP1R14D |
|  |  |  |  |  | DGAT2L7P |
|  |  |  |  |  | AGXT |
|  |  |  |  |  | C15orf38-AP3S2 |
|  |  |  |  |  | GOT1L1 |
|  |  |  |  |  | FAM47B |
|  |  |  |  |  | PLEKHG6 |
|  |  |  |  |  | KRTAP10-4 |
|  |  |  |  |  | JAK3 |
